# Evaluating the Resources and Need for a Lung Cancer Screening Pilot Program in Maryland

**DOI:** 10.5888/pcd15.170434

**Published:** 2018-07-12

**Authors:** Lisa D. Gardner, Cindy Domingo, JoAnn Johnston, Holly Harshbarger, Ken Lin Tai

**Affiliations:** 1 Center for Cancer Prevention and Control, Prevention and Health Promotion Administration, Maryland Department of Health, Baltimore, Maryland; 2 Department of Epidemiology and Public Health, University of Maryland School of Medicine, Baltimore, Maryland

**Figure:**
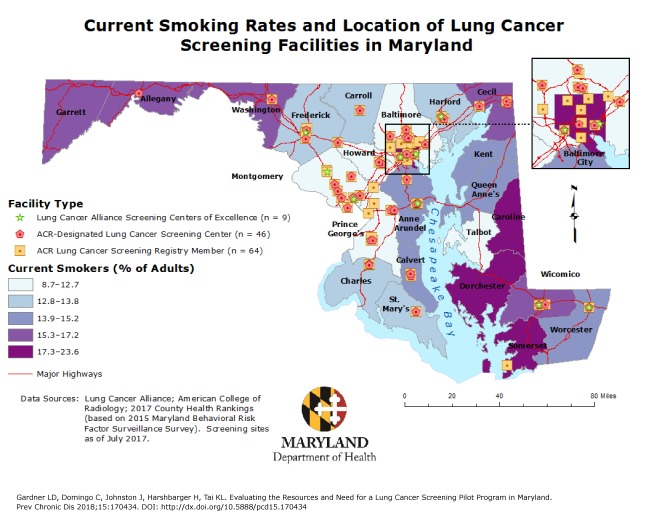
This map displays the locations of lung cancer screening facilities in Maryland and the estimated target population for lung cancer screening in each jurisdiction, based on current smoking rates (for 2015). Each location may have 1, 2, or all 3 types of screening facilities. Of the 119 screening facilities, 68 were unique. This map informed the Maryland Department of Health’s lung cancer screening pilot program by providing estimates of the eligible population for lung cancer screening by jurisdiction and showing areas with existing resources for lung cancer screening. Map created by Lisa D. Gardner on July 26, 2017. Abbreviation: ACR, American College of Radiology. **Table:** 

County	Smoking Rate, %	Lung Cancer Alliance Screening Center of Excellence, n	ACR-Designated Lung Cancer Screening Center, n	ACR Lung Cancer Screening Registry Member, n
Allegany	17.19	0	1	0
Anne Arundel	14.03	1	3	4
Baltimore	12.72	0	4	9
Calvert	14.39	0	2	2
Caroline	17.23	0	0	0
Carroll	12.99	0	2	2
Cecil	15.56	0	3	4
Charles	13.80	0	2	2
Dorchester	17.54	0	0	0
Frederick	12.95	1	2	4
Garrett	15.38	0	0	0
Harford	13.70	1	3	3
Howard	10.03	0	2	2
Kent	14.38	0	0	0
Montgomery	8.70	2	8	9
Prince George’s	12.62	0	5	8
Queen Anne’s	13.94	0	0	0
St. Mary’s	13.83	0	1	1
Somerset	20.31	0	0	1
Talbot	12.54	0	0	0
Washington	15.67	0	1	1
Wicomico	15.47	1	2	2
Worcester	14.64	1	1	1
Baltimore City	23.64	2	4	9

## Background

Lung cancer is the leading cause of cancer death in Maryland, and it is the third most common cancer after female breast and prostate cancers (1). In 2013, more than 2,600 Marylanders died of lung cancer (1). Most lung cancer cases are diagnosed after metastasis, so the 5-year survival rate is low at 18.1% (2). Finding lung cancer early drastically improves survival; the 5-year survival rate for localized lung cancer is 55.6% (2). Cigarette smoking is the primary risk factor for lung cancer. In 2015, 15.2% of Maryland adults (aged ≥18) were current smokers (1).

In 2011, the National Lung Screening Trial showed that annual screening with low-dose computed tomography (LDCT) in high-risk individuals reduced mortality from lung cancer by 20% (3). As a result, the National Comprehensive Cancer Network recommends annual LDCT screening of asymptomatic high-risk individuals, defined as the following: 1) adults aged 55 to 74 with a smoking history of 30 pack-years or more and smoking cessation for less than 15 years or 2) adults aged 50 or older with a smoking history of 20 pack-years or more and at least 1 additional risk factor (eg, cancer history) other than second-hand smoke (4). The US Preventive Services Task Force also recommends annual LDCT screening for lung cancer among certain high-risk individuals.

Cancer prevention and control under Maryland’s Cigarette Restitution Fund Cancer Prevention, Education, Screening and Treatment Program (CRF-CPEST) is a high priority for the Maryland Department of Health (1). The goal of CRF-CPEST is to reduce cancer mortality and morbidity rates in the state through implementation of community-based programs to prevent, detect, and/or treat cancer early. CRF-CPEST programs provide community education and outreach, funding to assist uninsured and underinsured Marylanders in obtaining access to cancer screenings, and case management to ensure linkage to screening, diagnosis, and treatment. Because lung cancer is 1 of 7 cancers targeted by CRF-CPEST, we evaluated the resources and need for establishing a pilot program for lung cancer screening in Maryland.

## Methods

Given that cigarette smoking is the main risk factor for lung cancer, we estimated smoking rates by jurisdiction (23 counties and Baltimore City) in Maryland to determine areas with the highest rates. Smoking rates from 2015 were estimated according to a statistical model that combined information from the Behavioral Risk Factor Surveillance System and the National Health Interview Survey to correct for nonresponse and under-coverage bias; this model is enhanced in small areas by borrowing information from similar areas nationally (5).

We identified lung cancer screening facilities in Maryland by using 3 publicly available sources: 1) the American College of Radiology’s (ACR’s) Lung Cancer Screening Registry, which is approved by the Centers for Medicare and Medicaid Services and enables providers to meet quality reporting requirements for receiving Medicare payments for lung cancer screening with LDCT; 2) the ACR Lung Cancer Screening Center designation, which identifies facilities that have achieved ACR accreditation for computed tomography in at least the chest module and provide safe, effective diagnostic care for those at high risk for lung cancer; and 3) the Lung Cancer Alliance’s Screening Centers of Excellence, which are organizations identified as being committed to responsible, high-quality screening practices. We then geocoded and mapped these facilities in ArcGIS version 10.4 (Environmental Systems Research Institute, Inc) to determine jurisdictions that had lung cancer screening capabilities.

## Findings

The map shows the percentage of adults who were current smokers in 2015 by jurisdiction. These percentages vary considerably from the state percentage (15.2%), ranging from 8.7% to 23.6%. Smoking rates were highest in Baltimore City, the lower Eastern Shore (Caroline, Dorchester, Somerset, and Wicomico counties), western Maryland (Allegany, Garrett, and Washington counties), and Cecil County. Only 2 counties (Montgomery and Howard) meet the 2020 Healthy People objective (12.0%) for reducing tobacco use among US adults (6).

As of July 2017, 119 lung cancer screening facilities were identified in Maryland: 1) 64 ACR Lung Cancer Screening Registry facilities, 2) 46 ACR-Designated Lung Cancer Screening Centers, and 3) 9 LCA Screening Centers of Excellence. Each facility is equipped to screen for lung cancer per ACR guidelines. The distribution of LDCT screening facilities reflects Maryland’s population density, with highly populated areas (eg, the Baltimore–Washington metropolitan area) having more facilities. Major highways are also displayed to demonstrate the accessibility of each facility to residents throughout the state.

## Action

The Maryland Department of Health’s CRF-CPEST Program initiated a lung cancer screening pilot program in fiscal year 2018. The pilot’s goal is to assist uninsured and underinsured high-risk Marylanders in obtaining access to life-saving screening, diagnostic, and treatment services for lung cancer. In the 5-year period from 2010 through 2014, 11 of 24 Maryland jurisdictions had significantly higher lung cancer mortality rates than the state rate of 43.1 per 100,000 (1). This pilot will help diagnose lung cancer cases at an earlier stage, improving survival and decreasing mortality. The pilot will also establish a working model for other CRF-CPEST programs that plan to implement lung cancer screening.

This map informed leadership of the Maryland Department of Health of the estimated eligible population for lung cancer screening (ie, percentage of adults who are current smokers) by jurisdiction and showed areas with resources for lung cancer screening. Although current smoking rates do not reflect the entire population at high risk for lung cancer, these rates are the only reliable risk data available. Because they exclude former smokers, the rates likely underestimate the population at high risk for lung cancer.

Eleven programs from 10 jurisdictions submitted applications to CRF-CPEST to participate in the pilot program. Selection of pilot participants was made according to the need and current resources of each jurisdiction, as demonstrated in our map, as well as their responses to an interview designed to assess readiness to implement lung cancer screening. Six programs (Allegany, Baltimore, Cecil, Harford, and Wicomico counties, and MedStar Health in Baltimore City) were selected to participate in the lung cancer screening pilot program for fiscal year 2018. All applicants, regardless of pilot program participation status, were extended invitations to attend training sessions throughout fiscal year 2018.

This map was also used to identify jurisdictions in which the prevalence of current smoking was higher than the state rate and that lack LDCT screening facilities but are interested in offering lung cancer screening services to their communities. These jurisdictions will be provided with technical assistance to ensure that lung cancer screening can be implemented in future years.
